# Interprofessional Paediatric High-Fidelity Simulation Training: A Mixed Methods Study of Experiences and Readiness among Nursing and Medical Students

**DOI:** 10.3390/nursrep14010044

**Published:** 2024-03-07

**Authors:** Helmut Beichler, Simone Grandy, Silke Neumaier, Anneliese Lilgenau, Hannah Schwarz, Michael Wagner

**Affiliations:** 1 Vienna Healthcare Group, School of Nursing, University of Applied Sciences FH Campus Vienna Floridotower Campus, 1210 Wien, Austria; silke.neumaier@akhwien.at (S.N.); anneliese13@me.com (A.L.); 2 Department Nursing Science, University of Applied Sciences FH Campus Vienna, 1100 Wien, Austria; simone.grandy@fh-campuswien.ac.at; 3 Comprehensive Center for Pediatrics, Division of Neonatology, Intensive Care Medicine and Neuropediatric, Medical University of Vienna, 1090 Wien, Austria; hannah.schwarz@meduniwien.ac.at (H.S.); michael.b.wagner@meduniwien.ac.at (M.W.)

**Keywords:** high-fidelity simulation training, paediatric, interprofessional learning, collaboration

## Abstract

Background: Training in communication skills between nursing and medical students during interprofessional paediatric emergency simulation training represents a significant aspect of safe patient care. Evidence highlights that poor communication in paediatric emergency scenarios jeopardises patient safety. Through realistic simulations, students practice the communication strategies of crisis resource management (CRM), such as “closed-loop communication”, “speaking up”, and “team time-out”. Aims: In this study, we aimed to evaluate the impact of interprofessional simulation on enhancing teamwork and communication skills. Additionally, we sought to assess the occurrence of contexts for the three CRM communication strategies during simulations. Methods: Employing a mixed methods research design, the analysed students completed pre- and post-simulation online questionnaires. To measure attitudes towards interprofessional collaboration, we used the German version of the University of West England Interprofessional Questionnaire (UWE-IP_german), and to measure interprofessional attitudes, we used the Greifswald Questionnaire for the Measurement of Interprofessional Attitudes (Greif Mie), also in German, for both pre- and post-simulation. For qualitative video analysis, we utilised Grounded Theory Methodology (GTM). Results: Following simulation training, we observed a significant improvement (*p* > 0.001) in the subscale “attitude towards interprofessional learning” of the UWE-IP_german among nursing and medical students. Medical students consistently exhibited a significantly (*p* < 0.001) more positive attitude towards other professionals in both the pre- and post-simulation assessments. Overall, all the students expressed satisfaction with the interprofessional simulation training. In the qualitative selective coding process, the central phenomenon “participation” could be identified in the coding paradigm. Conclusion: This study presents substantial evidence of the learning impact of interprofessional paediatric simulation training on nursing and medical students.

## 1. Introduction 

Emergency situations affecting critically ill paediatric patients are frequent occurrences in clinical settings [1]. These settings often present challenging conditions for patient care due to limited space, noise, constraints on resources, and the presence of the distressed family members of a critically ill child [2].

Interprofessional paediatric simulation refers to an educational approach in which members from different disciplines (such as physicians and nurses) collaborate to solve complex problems or scenarios likely to arise in real work environments [3]. These simulations are carried out in controlled settings, integrating high-fidelity simulators that allow participants to gain hands-on experience and enhance their skills without jeopardising patient safety [4].

Paediatric intensive care units (PICUs) are clinical areas involving a diverse array of multidisciplinary healthcare professionals including physicians, nurses, and other healthcare professionals [5,6]. Effective professional teamwork during emergency management increases patient safety and reduces error rates and mortality [7]. Simulations are designed to replicate real emergency settings as authentically as possible [8,9,10]. The realistic construction of case studies, simulated using high-fidelity mannequins, forms the basis for crisis resource management training [11,12].

The project “Interprofessional Team Simulation Training in Education”, a cooperative project between the Medical University of Vienna and the Nursing School at Floridotower Campus, was established in Vienna in 2016. Since 2022, interprofessional simulation training, involving students from both the Medical University of Vienna and Campus Floridotower, a site of the FH Campus Wien University of Applied Sciences, has taken place in the Department of Paediatrics and Adolescent Medicine at the Medical University of Vienna. The focus of these interprofessional simulation sessions, involving students from both institutions, is on training in the acute paediatric setting using a high-fidelity simulator (SimBaby from Laerdal [13]. These training sessions, led by lecturers from both professional backgrounds, encompass scenarios like acute bronchiolitis and dealing with a dehydrated infant. We focus on the training of human factors as well as communication strategies (closed-loop communication, speaking up, and team time-out), within the framework of crisis resource management (CRM) guidelines [14,15,16]. CRM is defined as the general management and prevention of incidents [17]. CRM originally comes from aviation, wherein it was known as Cockpit Resource Management and later as Crew Resource Management, and was adapted for medicine by David Gaba in the form of anaesthetic crisis resource management (ACRM) [18]. The aim of CRM is to help an individual and their team to reduce the rate of complications and incidents during an emergency (the preventive approach) and to act more effectively and without error in the management of emergencies (the reactive approach) [19,20]. 

Errors in communication during an emergency simulation are identified with the goal of optimising communication strategies during an emergency [21,22]. The overarching aim of these simulations is to minimise human error and enhance patient safety [23]. 

Research suggests that about 70 percent of incidents in emergencies with poor outcomes stem from “human factors” rather than a lack of expertise or knowledge on the part of the involved professional groups [24,25]. For this purpose, individuals are trained in three communication strategies: closed-loop communication, team time-out with ten seconds for the next ten minutes (10-for-10), and speaking up. Closed-loop communication means that a requested action in the emergency setting should be repeated verbally by the person addressing before it is carried out [26,27]. The 10-for-10 team time-out is intended to allow all team members involved in an emergency to take a short break while life-sustaining measures continue to be taken [14]. It serves as a summary of the current situation, including a reflection on all interventions [22]. This practice enables a team to regather and thus achieve more effective results [12]. Speaking up is proposed to offer all disciplines involved in an emergency—irrespective of their professional status or hierarchical anchoring—the ability to voice concerns about treatment in terms of patient safety as well as quality of care if they perceive that a potentially risky or erroneous action has been taken by other actors [28,29]. 

**Figure 1:**
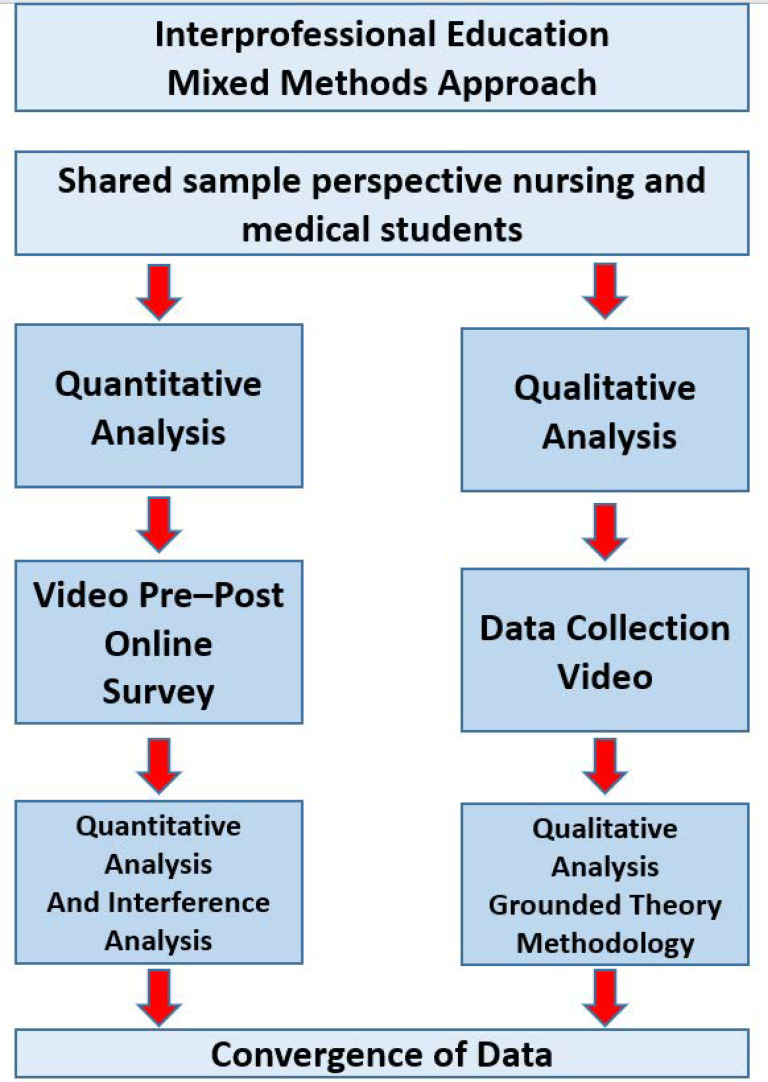
Mixed methods procedure.

The aim of this study was to assess the impact interprofessional simulation has on enhancing the teamwork and communication skills of the participating students. Additionally, the occurrence of the context of different communication strategies during simulation was analysed qualitatively and quantitatively (Figure 1).

## 2. Materials and Methods

### 2.1. Design

In a mixed methods design (Figure 1), data were collected through qualitative and quantitative video analysis of simulation recordings intended for debriefings and via quantitative pre–post surveys. During the simulation training, two groups were formed, each consisting of four students, with two medical students and two nursing students forming one team in a paediatric emergency scenario. 

The scenarios were planned based on theory by analysing the students’ needs and the defined learning objectives. Furthermore, suitable tasks were developed, including with regard to the complexity of a case. In addition, the process was planned and reviewed. When planning the scenarios, it was important to define the learning objectives in advance, as the implementation of the training depended on this and influenced the process of developing criteria for the debriefing.

Altogether, 30 simulation scenarios were conducted. Each scenario was recorded and then reflected on with students in a structured, collaborative debriefing session. 

**Figure 2:**
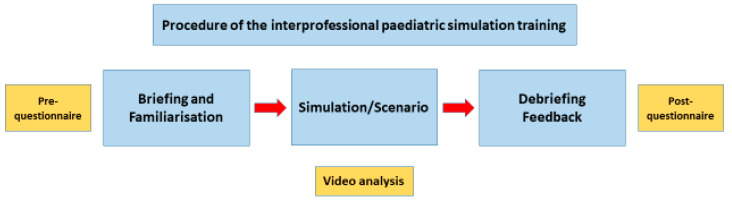
Procedure used to acquire the interdisciplinary simulation and survey data.

The interdisciplinary simulation training (Figure 2) consisted of briefing, simulation, and debriefing phases. 

### 2.2. Participants and Setting 

Nursing and medical students were enrolled in the simulation training from their 3rd year onwards. A total of 65 participants engaged in the qualitative video analysis, 29 of whom were medical students (10 males and 19 females, with an age range of 21–29 years). The nursing students (total n = 36) were divided into 29 females and 7 males, with an age range of 23–40 years. A total of 108 medical and nursing students took part in the quantitative pre–post surveys. Among these, 76 students (41 from medicine and 35 from nursing analysed in the quantitative analysis) completed the questionnaire, making it possible to clearly assign their pre- and post-survey data. These n = 76 cases were used for the following analysis (response rate = 70%).

Two weeks before the training commenced, the students received instructional documents via email covering the basics of paediatric life support as well as the fundamentals of paediatric emergency management and human factors. 

**Figure 3:**
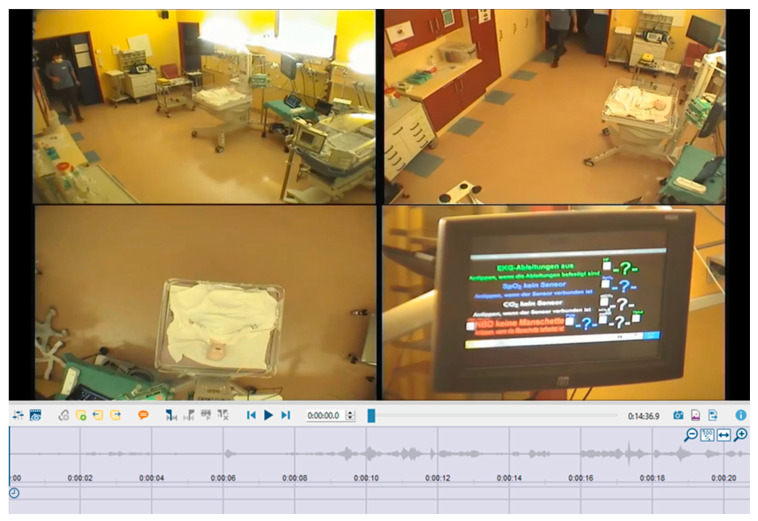
Simulation setting and simulation videos processed using MAXQDA software, 2023, Germany.

The simulation centre of the Department of Paediatrics and Adolescent Medicine at the Medical University of Vienna is equipped with state-of-the-art technology and simulation software (Figure 3). 

### 2.3. Quantitative Procedure 

#### 2.3.1. Data Collection and Questionnaire

The quantitative data collection was facilitated through an online questionnaire on the online platform Unipark. The questionnaire consisted of eight blocks. The initial block included information on data protection, including informed consent, and assignment to the pre- and post-surveys with a self-selected letter code. Sociodemographic information was collected in block two. Blocks three to six encompassed the validated University of the West of England Interprofessional Questionnaire (UWE-IP) in German [30,31]. The UWE-IP_german consisted of four subscales. Block three included the Communication and Teamwork subscale with nine items; block four covered the Interprofessional Learning subscale with nine items; block five encompassed the Interprofessional Interaction subscale with nine items; and block six included the Interprofessional Relationships subscale with eight items. Items were measured on 4-point (Communication and Teamwork subscale) and 5-point (all other subscales) Likert scales. 

Block seven consisted of items 1–10, corresponding to the Attitude towards the Other Professional Group subscale of the Greifswald Questionnaire to Measure Interprofessional Attitudes (Greif Mie), with ten items rated on a 5-point Likert scale [15,32]. Block eight consisted of five questions about the evaluation of the interprofessional course. Quantitative data analysis was conducted using SPSS Statistics (IBM SPSS Statistics 27).

In addition, we conducted a quantitative secondary data analysis of 18 debriefing videos involving the enumeration of the human factors (team time-out, closed-loop communication, and speaking up) and their occurrences. 

#### 2.3.2. Quantitative Data Analysis

As the metric scale did not meet the requirement of a normal distribution, the Wilcoxon test was utilised to evaluate significant pre- and post-testing differences. Effect size was calculated according to Field (2013) [33] as the value z divided by the square root of all observations. This value can range between 0 (no effect) and 1 (maximum effect). According to Cohen (1992) [34], a value of r = 0.1 corresponds to a weak effect, a value of r = 0.3 corresponds to a medium effect, and a value of r = 0.5 corresponds to a strong effect (Field 2013: 227).

### 2.4. Qualitative Procedures 

#### 2.4.1. Data Collection

Among the simulation scenarios generated during the interprofessional simulation training, 18 scenarios, which were recorded on video, were made accessible for secondary data analysis with the consent of the participating students. The research team imported all the video footage (18 individual scenarios, amounting to approximately 6 h of video footage) into MAXQDA software during the fall of 2022 and reviewed it together. Each of the 18 scenarios was recorded simultaneously from four different perspectives (the entire room from the left, the entire room from the right, an overhead view of the high-fidelity mannequin, and a view of the monitor), as illustrated in Figure 3. Each scenario was imported into MAXQDA 2022 as a video with a pictorial four-way split.

#### 2.4.2. Data Analysis

The context of the trained communication strategies underwent qualitative analysis following Grounded Theory principles outlined by [35] using MAXQDA 2022.

The video and text passages from the data material were documented for inter-subjective comprehensibility, along with the corresponding categories. Each video was screened, and the communication strategies (closed-loop communication, speaking up, and team time-out) were marked and coded according to each context on the timeline. Anchor quotes were transcribed from individual video sequences into textual form.

In the open coding process, codes for different contexts in emergency situations including the communication strategies were explored. This stage progressed to categorising codes and sub-codes, enhancing abstraction levels.

Axial coding focuses on establishing connections between categories. It places an axis in the centre with the central phenomenon. The axis is between the causal conditions of the phenomenon and the consequences. The link between the causal conditions and consequences is the action strategies. The phenomenon is flanked by certain contextual conditions/intervening conditions (temporal and local conditions, social conditions, cultural environment, and individual biography). Throughout this process, theoretical memos documenting the analysis process were created. The final phase of the selective coding of the central phenomenon could be identified and completed with the core categories on the axis.

### 2.5. Ethical Considerations

The simulation study received an exempt status from the institutional review board. No identifiable personal data were collected throughout the study. All basic principles of the Helsinki Declaration were applied. Participation in the simulation was voluntary for the students, and data analysis was conducted anonymously. Informed consent was obtained from participating students specifically for the secondary analysis of the videotaped simulations, typically utilised solely for debriefing purposes. The students were not graded for their participation in the interprofessional training and retained complete freedom to withdraw their consent at any point.

## 3. Results

### 3.1. Quantitative Results

#### 3.1.1. Pre–Post Online Surveys

Between February 2022 and January 2023, a total of 30 simulation scenarios were conducted as part of the interprofessional simulation training, reaching n = 108 nursing and medical students. Of these, n = 76 students completed the pre- and post-online surveys, resulting in a response rate of 70%. Case assignments were established through pre-coding by using the first letters of the participants’ fathers’ and mothers’ names. 

The demographic characteristics of the nursing and medical students surveyed showed that there was a significant majority of females (72%) compared to males (28%). Most of the medical students were between 24 and 26 years old and in their fifth academic year. The nursing students were between 21 and 23 years old, and nearly all of them were in their third academic year. On average, the nursing students were slightly younger than their medical counterparts, and about three-quarters of the students were female (Table 1).

Table 2 provides a concise summary of all five scales used to measure possible outcome change after participation in the interprofessional simulation. As none of the scales exhibited a normal distribution according to the results of the Kolmogorov–Smirnov test, the nonparametric Wilcoxon test was applied to assess the significance levels of the pre- and post-simulation data [33].

Overall, the students provided highly positive ratings across all five scales, as Table 2 shows, and both cohorts showed a highly significant improvement in attitude towards interprofessional learning after the simulation. (The scale is polarised; so, lower scores mean more positive attitudes.) The effect of this improvement was strong for both groups (for medical students, r = −0.52; for health and nursing students, r = −0.67). 

After the simulation, the medical students also showed a significant improvement in scale scores related to “Attitude towards Interprofessional Interaction”, assessment of own “Interprofessional Relations”, and “Attitude towards Other Professional Group”. In contrast, the group of healthcare and nursing students showed a significant improvement in the assessment of their skills in “Communication and Teamwork”.

The attitude towards the other professional group (rated on a scale of 0–100) was rated as very favourable in both groups. Interestingly, the difference between the groups was highly significant for both the pre-value (U = 385, z = −3.530, *p* < 0.001, and n = 76) and the post-value (U = 417.5, z = −3.001, *p* = 0.003, and n = 74), according to the results of the Mann–Whitney U test. This finding suggests that, compared to nursing students, medical students exhibited a significantly more positive attitude towards the other professional group both before and after the simulation. However, as previously mentioned, nursing students significantly improved with respect to this value as well.

The interprofessional simulation training received highly positive feedback from both medical and nursing students, with an overall recommendation rate of 99%. Notably, 95% of all the students rated the training as “excellent” or “good.” In general, most of the students felt that the available time for the simulation and debriefing was well balanced, with 25% of medical students wishing for slightly more time for the simulation compared to only 6.7% of the nursing students. The complexity of the case scenarios seemed appropriate for many students in both groups.

#### 3.1.2. Quantitative Video Analysis

**Figure 4:**
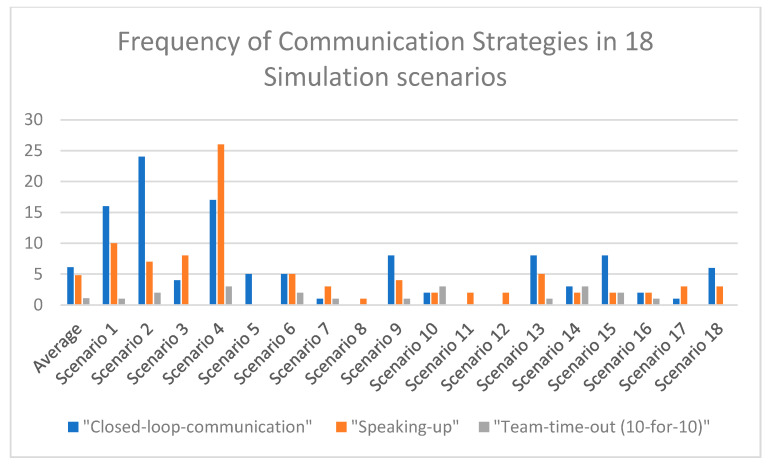
Frequency of communication strategies.

The quantitative analysis of a total of 18 simulation videos, for which consent for secondary data analysis was obtained from n = 65 participating students, revealed that the communication strategies taught in the course (10-for-10, closed-loop communication, and speaking up) were employed to varying degrees by the interdisciplinary teams. As illustrated in Figure 4, the student groups employed “closed-loop-communication” an average of six times per scenario (spanning approximately 15–20 min), whereas in certain scenarios (for example, in scenarios 8, 11, and 12), this communication strategy could not be observed even once, while in other scenarios, such as in scenario 2, Closed Loop Communication was used a total of twenty-four times. 

“Speaking up” was observed about five times per scenario (on average), showing a variance similar to that of “Closed Loop Communication”. Scenario 4 clearly stood out, with a total of twenty-six observations of “Speaking Up”; only scenario 5 did not have a single occurrence of this strategy recorded. The strategy “10 s for 10 min” could be observed an average of one time per scenario. 

Surprisingly, certain scenarios were conducted without a single team time-out. In scenarios 3, 5, 8, 11, 12, 17, and 18, no team time-outs were observed during the video analyses by the research team. Conversely, in scenarios 2, 4, 6, 10, 14, 15, and 16, the strategy was applied 2–3 times. Across all 18 scenarios, no instances had more than three team time-outs recorded.

Overall, approximately 40% of the 18 observed interprofessional simulation training sessions lacked a team time-out (10 s for 10 min) during the simulation. In 17% of the observed 18 simulation scenarios, “Closed Loop Communication” did not occur once, whereas only one scenario lacked instances of “speaking up”. Based on this evaluation, the recommendation for future interprofessional simulation training would be to proactively emphasise the importance of the “team-time-out” and “closed loop communication” strategies in advance.

Additionally, an exploratory assessment was conducted to ascertain whether the frequency of the observed communication strategies influenced scenario quality. A descriptive evaluation of quality was conducted using a predefined three-tiered scale (where 1 corresponds to very good, 2 corresponds to moderate, and 3 corresponds to poor). The assessment criteria were defined based on the presence of a clear leader, a structured sequence of the emergency, the number of communication strategies employed, and the adherence to evidence-based emergency guidelines provided by the European Resuscitation Council. Nine videos (50%) were rated independently from each other by two simulation instructors as very good, five videos (28%) were determined to be moderately good, and four videos (22%) were assessed as being poor. Regarding the closed-loop communication strategy, there was a tendency observed wherein in scenarios rated higher in quality, communication strategies tended to be used more frequently (Table 3). In scenarios rated as “very good” by the instructors, “closed loop communication” occurred an average of 7.3 times; whereas, in scenarios rated as “moderate”, this strategy was observed an average of 6.3 times. In scenarios rated as “poor”, the “closed loop communication” strategy was observed merely three times (on average) during the simulation scenario.

### 3.2. Qualitative Results

**Figure 5:**
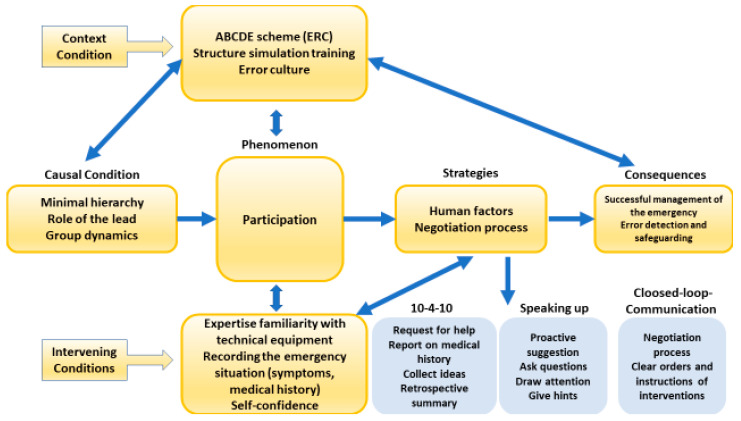
Simulation situation with the central phenomenon participation.

After completing the open- and axial coding processes, three axial coding paradigms were established. The final paradigm involved selective coding, pinpointing the central phenomenon of “participation”, and completed using the core categories on the axis (Figure 5). The causal conditions with a low hierarchy, the role of the lead, and the group dynamics during the paediatric interprofessional emergency scenario are on the axis of the selective coding paradigm.

The strategies encompassed three communication aspects: (1) closed-loop communication, (2) speaking up, and a (3) team time-out of ten seconds for the next ten minutes. The consequences were identified as the appropriate management of the whole emergency, including safeguarding and error detection. 

The context conditions were delineated by the ABCDE algorithm (A = Airway, B = Breathing, C = Circulation, D = Disability, and E = Exposure) as per the European Resuscitation Council (ERC) guidelines, the simulation training procedure, and the individual as well as collective error culture among nursing and medical students. 

Intervening conditions centred on the phenomenon, encapsulating the expertise of the participating nursing and medical students, their familiarity (ensured via familiarisation) with the technical devices, and their self-confidence. 

The context conditions are associated with the causal conditions and their consequences. The foremost impact was the successful emergency management of a critically ill paediatric patient, error detection in accordance with speaking up as a strategy, and safeguarding focused on patient safety. 

The intervening conditions, including expertise and familiarity with technical equipment, recording the emergency, and the self-confidence of nursing and medical students, are closely related to the communication strategies and the central phenomenon. These conditions significantly contribute to the outcome of the successful management of an emergency, error detection, and safeguarding measures.

#### 3.2.1. Central Phenomenon: Participation 

Participation in interprofessional teams is of crucial importance in the emergency setting, as all interprofessional team members should be equally involved in managing a situation. Participation refers to the active involvement of the medical and nursing students in order to promote the exchange of expertise appropriate for the planned interventions and to optimise communication. The crucial factors for active participation included the prevalent presence of a low hierarchical structure within the team, which could be observed in almost all scenarios; the establishment of a lead before starting the simulation; and group dynamics. This was characterised by polite, structured, clear, and unambiguous communication. The following dialog depicts when a lead clearly emerges during a scenario:

*The mother (played by a teacher) of the sick infant appeared very agitated, nervous, and worried. She spoke in a shaky voice. The team’s pre-designated lead remains professional, keeps calm, and asks specific questions about symptoms as part of the patient history. All team members are addressed by name when interventions are ordered. Specific actions and interventions are directed specifically to the individual, e.g., lead is a prospectively defined medical student and states “Please, bring the mother in the emergency room again, we still need her for the anamnesis.” (T3, SC1, 0.05.52–0.06.44).*

Each team member’s role distribution was defined by the assumption of conventional positions, such as a leadership role with respect to the medical staff (nurses and physicians). 

This group dynamic was reinforced by time pressure, complexity, and pressure to make decisions while maintaining low error tolerance. These factors also had the effect of significantly increasing stress levels. It should also be emphasised that the lead function was assumed by both nursing and medical students, especially when the nursing students were already highly familiar with emergency scenarios. In addition, our analysis showed that nursing students usually had an advantage over medical students in terms of practical experience. This is understandable due to the extensive number of continuous internships within the nursing study program. Despite the existence of a lead, individual video sequences of the scenarios were observed to be chaotic, not very structured, and hardly participatory. The participants needed to communicate effectively with each other to share information, make decisions, and solve problems together. 

The intervening conditions are reciprocally related to the central phenomenon of participation and were identifiable by the students’ expertise in paediatric emergency management, especially with respect to the lead, as well as their familiarity with technical equipment, ability to record an emergency (regarding symptoms, medical history, etc.), and self-confidence. Intervening conditions were particularly evident due to the initial challenge in grasping an unanticipated paediatric emergency. In addition, time pressure, decision making and process flows, and the dynamic patient situation, including a variety of external influences, affected the situation. The external influencing factors encompassed a distressed mother or father of the paediatric patient. In addition, an overtaxed physician (medical student) or an ambitious, experienced nursing student could often be identified. In some emergency scenarios, a very heterogeneous group was revealed in terms of knowledge level and skills but also personal experience. The participants were challenged to work together to solve complex problems that required a variety of skills and competencies. By integrating the subject matter expertise of different professions, they were able to develop more comprehensive approaches to manage problems. However, in certain scenarios, participation involved interprofessional negotiations between medical and nursing students, encompassing discussions on diagnoses, working hypotheses, medication orders, and reference values for hemodynamic vital signs. The negotiation process was also related to issues such as medication and the calculation of infusions, a calculation made based on body weight. In particular, the negotiation process was initiated when the emergency response was chaotic and unstructured, no pre-defined lead was present or apparent, and the students had different levels of knowledge and skills. This was evident in the lack of working hypothesis as well as knowledge of the actual underlying problem of the critically ill child. Without a preliminary or potential differential diagnosis and a working hypothesis, there was a high risk of confusion among nursing and medical students, resulting in a situation where none of the team members knew what the appropriate course of action was, thereby limiting constructive proactive discourse, in which no joint outcome-based decision was reached. Individual team members were not responsive to each other. This became clear in individual video sequences, wherein unstructured, incoherent ideas, observations, and arrangements were put forward by various team members.

*The lead (medical student) shouted an order for the administration of an infusion in a non-targeted, uncoordinated manner whilst also failing to specify which member of the team he was asking to perform this task. “We are now giving 10 mL of infusion.”*

*One of the nursing students was confused but responded with “Ok, yes” and acknowledged the order, but the execution was not implemented (T3, SC2, 0.067–0.075).*

This video sequence appeared uncoordinated and chaotic as it progressed after missing the name of the infusion to administer, including the correct dose. Despite the uncoordinated situations in most scenarios, a minimal hierarchical structure and a high level of participation were observed.

*A nursing student shouted into the room “Frequency and pulse are 191.”*

*The lead (medical student) did not react to this and asked the question: “What does the ECG look like?” Another nursing student responded to the infusion order from the dialog before and said “10 mL.”*

*Another nursing student called into the emergency room: “How is the child breathing?”*

*The lead (medical student) ordered: “Then we need more oxygen. That is already resuscitated.”*

*The nursing student responded to the order for oxygen: “What does she have?”*

*The nursing student responded to the oxygen order: “I’m turning up the oxygen to 9 littres.”*

*Finally, this sequence showed another nursing student with 20 mL of infusion administration (T9, SC1, 0.07.14-0.08.12).*

The dialogue made it clear that the ABCDE algorithm of the European Resuscitation Council (ERC) was not applied consistently and stringently and that the individual team members concentrated on individual aspects in an unstructured manner. 

Students in both fields of study questioned orders they were given and gave indications of altered hemodynamic vital signs that remained inconsequential. 

The students stared at the monitors during critical situations involving vital signs indicative of life-threatening situations and appeared unable to act. These learning observations could be related and constructively reflected upon in the patient safety debriefings. The analysis of the videos clearly showed very different levels of know-how and knowledge in both student groups, which were characterised by a lack of communication.

#### 3.2.2. Human Factors as Strategies

**Figure 6:**
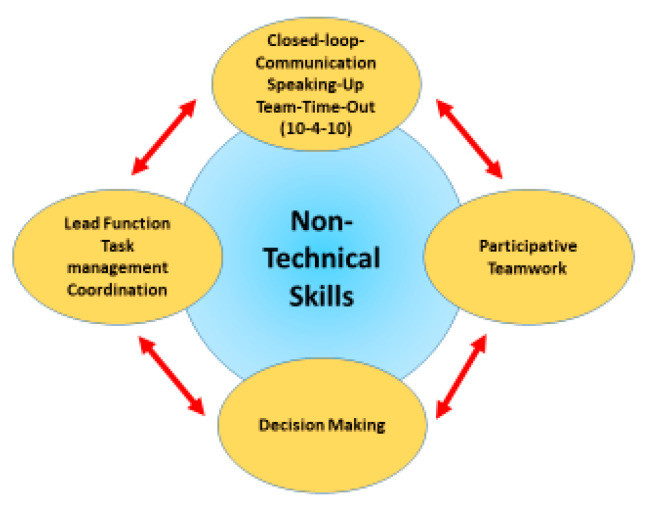
Human factors and non-technical skills in simulation training. (This graphic was produced by the authors.)

The human factors such as team time-out (10-for-10), closed-loop communication, and speaking up were identified as the basic strategies in the selective coding process. These strategies are closely intertwined with the intervening conditions, including empathy, confidence and expertise in emergency settings, and familiarity with emergency technical equipment and pharmacology. It was observed in the videos that the communication strategies were not always clearly distinguishable from each other. In some cases, the individual strategies were inter-related or complemented each other to achieve the desired outcome. The communication manifested both verbally and non-verbally and was characterised by awareness of the situation, the demand for teamwork, decision making, and task management and task distribution. Good teamwork was identified as an influencing factor for the communication strategies, as presented in Figure 6.

It was clearly shown that the team had to fit together and communicate with each other. These actions were also observed at the interfaces between the lead nursing and medical students and the parents of the critically ill child. 

Cognitive aspects related to individuals were evident in their deliberate focus on the emergency, the dynamics of the decision-making process, the establishment of priorities, and, notably, in assuming leadership roles. This allowed the students to recognise and largely avoid fixation errors. Requesting help by triggering the emergency alarm or calling in experts was mostly forgotten in the scenarios (presumably due to fixation errors and stress). 

The analysis also revealed a lack of communication. This was evident in some video sequences in which the team members almost neglected to engage in any communication among themselves whatsoever. The results clearly showed that the students were stalled in communication when they were very overwhelmed in dealing with the complex emergency situation. Lack of experience and inadequate medical knowledge were recognised as factors that contribute to both medical and nursing students becoming overwhelmed and further limited communication. In addition, different scenarios showed a different understanding of the profession, divergent goals and work processes, a lack of a mission and insufficient clarity regarding leadership, scarce resources, and an untargeted use of resources. 

#### 3.2.3. Team Time-Out 

In team time-outs, all team members listen briefly, all information is gathered, ideas are contributed, and any concerns are expressed. Then, a plan is made for the next 10 min. Resources are also distributed during this process. The team time-out is identifiable by the aspects of calling for help, reporting the history of the critically ill child, collecting ideas from other team members, and retrospectively summarising the critical situation of the paediatric emergency patient by referring to the video material. 

In the videos, it could be observed that during these team time-outs, every person was asked to express all their ideas, considerations, and hypotheses. Likewise, in this short time, a briefing including the summary of the interventions already implemented (mostly the updating of the ABDCE algorithm) as well as the prospective planning and consideration of future interventions. Evaluation on the impact of the interventions was also part of the team time-out. It was observed that each team member could initiate and co-participate in a team time-out without hierarchical constraints. Sometimes, the team time-out resulted in a mixture of the clarification of orders and the adjustment of already-set interventions, also constituting a negotiation process between medical and nursing students.

*Lead (medical student) ordered the nursing student to prepare an infusion: “Please first we prepare an infusion.”*

*The nursing student replied to this order and at the same time initiated the discussion for an update of symptoms that the nurse considered important:*

*“Yes, but did you hear what the mother said before: The child vomited and had diarrhea last night and since then the child has not eaten. Now we must think, what can this be? Do you understand?!” (T1, SC1, 0.08.46–0.08.56).*

Moreover, this dialogue highlighted the importance of understanding the critically ill child’s condition and establishing a diagnosis before ordering a nursing student to administer infusions. Different video sequences could be identified with the evaluation of the ABCDE algorithm as a structure for the team time-out. This result makes it evident that the ABCDE algorithm of the ERC was used as a default in the briefing by most of the students.

*Lead (medical student) initiates a team time-out to clarify the circulatory and respiratory situation: “We still have an airway obstruction. There is a cough present. What does the team say about the symptomatology? Let’s go through the ABCDE-algorithm together again.*

*We have an A (Airway) problem, B (Breathing), the breathing itself fits.*

*C (Circulation, the recapillarisation time was collected and found to be slightly increased.”*

*Furthermore, concrete questions were asked to the team on how the situation can be managed: “What does the team think? Does anyone else on the team have any ideas? How can we make it easier for her to breathe? How can we force coughing and suctioning to improve breathing?”*

*After that, the evaluation was set to the whole team: “Let’s evaluate further.”*

*“What do we do next? Who has an idea?*

*“What is our working diagnose?” (T1, SC2, 0.04.26–0.04.54).*

#### 3.2.4. Closed-Loop Communication 

Closed-loop communication was identified as an essential link between the lead and the team. It became particularly clear in the analysis that the entire simulation situation ran more orderly through a complete closed-loop communication process. Nevertheless, it could also be determined that some essential rules were not always observed in terms of safe communication. 

This primarily occurred because either the lead did not explicitly and clearly assign instructions to the person involved or the nursing student did not offer feedback or confirmation, which could involve repeating the intervention. In addition, it was noticed that some medication orders were shouted incompletely into the emergency room. 

In these video sequences, a high degree of stress, triggered by inexperience in paediatric emergency situations, as well as a lack of knowledge of basic life support guidelines and pharmacological aspects, was identified.

Closed-loop communication was used as a negotiation process in the scenarios:

*Lead as a medical student is unsure about the dosage of Adrenaline: “How often can you give epinephrine?” Nursing student: “Every 3–5 min or? What do you say?” Other nursing student: “Then time interval to give epinephrine is still too short.” Lead: “I prepare it already.” (T2, SC2, 0.09.04–0.09.10).*

*Also, the combination of closed-loop-communication with speaking up became visible.*

*Lead (medical student) followed the ABCDE algorithm with specific questions. Breathing check: is the child breathing? Is the child showing signs of life? I start with the emergency check and do the breath check. The child is moving; she is breathing. A directed call to a nurse follows: Please connect the ECG electrodes and mount the child.*

*The nursing student acknowledged the order and performed the order and reported back to the Lead: electrodes are connected*.

*This was followed by another order specifically to the next nursing student: Please prepare and place an indwelling vein catheter.*

*This nursing student also confirmed the order as understood, performed the implementation of the indwelling vein catheter, and reported back that the implementation was performed (T8, SC2, 0.034–0.042).*

*A medical student as lead ordered to a nursing student: Can you please count the respiratory rate?*

*Nursing student confirmed in the completed closed-loop-communication cycle the order: Yes, got it, I’ll do it (T1, SC2, 0.05.06–0.05.10).*

Closed-loop communication was initiated by both medical and nursing students. In the emergency scenario, closed-loop communication was demonstrated by the example of the ABCDE algorithm, in which the individual aspects were addressed and confirmed. 

Moreover, closed-loop communication was implemented to share responsibilities within the team and to ensure patient safety in the presence of high-potency medications (catecholamine and anti-arrhythmic drugs).

#### 3.2.5. Speaking Up

Within individual sequences, the paediatric emergency emerged as a dynamic working environment for nursing and medical students. Here, priorities needed constant adjustment in response to changes in the critically ill child’s condition. To capture these changes accurately and effectively, the immediate communication of observations from each team member, free from hierarchical restrictions, becomes essential. This could be analysed in most of the videos. 

Speaking up could be identified in the video analysis as both drawing attention to sudden changes and proactively suggesting a variety of interventions. Also, when speaking up, a negotiation process was repeatedly noted, where the nursing students had to assert themselves with respect to their observations in a discussion. 

Numerous suggestions for measures as well as orders for medication were proactively initiated by nursing students on their own and discussed in an interprofessional manner.

It could be ascertained in several videos that the nursing students had significantly more experience in paediatric care. Therefore, it is understandable that due to this knowledge advantage over the medical students, the nursing students had more confidence and knowledge to draw upon and thus initiate proactive suggestions.

*A nursing student questioned whether the breathing fits: Maybe the airway is really misplaced?*

*Lead (medical student) replied: No, the airways are open.*

*Nursing student answered: Yes, but maybe there is mucus in the lungs and that is why the airways are blocked. Shouldn’t we suction after all (T1, SC1, 0.06–0.09).*

An unexperienced physician or nurse who showed little experience in an emergency could become a risk for a paediatric patient. The scenarios showed a heterogeneous picture of experience and knowledge between nursing and medical students. Proactive ideas and interventions were proposed and implemented through the confident initiative of nursing and medical students. 

One nursing student independently made the following decision: *I am determining the recap time (T1, SC1, 0.05.36–0.05.40). Also asking specifically for orders. Nursing student: should we give oxygen? (T2, SC1, 0.02.32–0.02.35). The proactive suggestion to give medication to stabilise the heart rate showed to be speaking up strategies. Nursing student asked and made the proactive suggestion: should we give epinephrine? (T2, SC1, 0.07.49–0.07.52). In addition, raising awareness of altered hemodynamic. Nursing student: should we take blood pressure again? (T2, SC2, 0.01.44–0.01.46). Giving indications of changes, Lead: we need oxygen. Nursing: should we ventilate the patient with the respiratory bag? (T2, SC2, 0.01.46–0.01.51). This could be identified. Requesting the cardiac alarm as well as the optimal use of help also emerged as a significant aspect in speaking up. Nurse: should I call the cardiac alarm already? (T1, SC2, 0.10.18–0.10.21). Patient safety and avoiding injuries were at the forefront of these efforts*.

This dialogue highlights how the inexperience of the lead, being a medical student, blurred the roles between nursing students responsible for executing orders. This mixing occasionally led to unclear distinctions between their respective responsibilities.

The human factor strategies resulted in the successful management of the emergencies, error detection, and patient safety. Closed-loop communication was used as a strategy for supporting patient safety. Speaking up was used to detect and avert errors. The team time-out was used to cope with the emergency, in which the team members made an update and planned further steps ahead. The determination of the lead role in various forms also contributed to the successful management of an emergency.

## 4. Discussion 

This mixed methods evaluation study, incorporating both a quasi-experimental and qualitative design, focused on “Interprofessional High-Fidelity Simulation in Paediatric Emergencies with Nursing and Medical Students.” It investigated the impact of interprofessional simulation on enhancing communication and teamwork, interprofessional learning, and interprofessional interactions, relationships, and attitudes towards the other professional group.

Mohaupt et al. (2012) [36] confirmed that the interactive element of learning is a central aspect of interprofessional education, in which participants should learn with, from, and about each other. King et al. (2016) [37] validated the effectiveness of high-fidelity simulation training in nursing education.

In principle, the human factors examined in this study are based on the 15 guiding principles of crisis resource management (CRM) [26]. The human factors are divided into five sub-areas (Figure 6). The non-technical skills as communication strategies represent the links between the individual aspects of the human factors. The main focus was on the communication strategies (closed-loop communication, speaking up, and team time-out). 

Each individual communication strategy is relevant and important both on its own and in combination. This was shown in the analysis of the contexts in which the closed-loop, speaking up, and team time-out communication strategies were used.

The descriptive quantitative evaluation of the videos focusing on the frequency of the three communication strategies revealed significant variance across individual scenarios. This observation is intriguing, considering that all the students received identical guidance, emphasising the significance of the human factor during the briefing. Delisle et al. (2016) [21] reported that 400,000 reversible deaths per year are caused by medical errors. Wai et al. (2017) [38] described the individual cognitive factors, such as the willingness to prioritise situational awareness or the ability to multi-task, including cooperative team factors, which significantly contribute to the overall effectiveness of an emergency response. This is in line with the research by Jowsey et al. (2020), [39] who asserted that the cognitive, psychological, physical, and social areas of human behaviour can be improved, particularly through interprofessional learning. Moreover, performativity, identity formation, and professionalism are developed in the context of clinical simulation training [4,13]. In addition, our study confirmed that there was a generally positive attitude towards interprofessional learning among both medical and nursing students. Jakobsen et al. (2018) [40] similarly confirmed a significant improvement in attitudes towards interprofessional learning and towards the other professional group and emphasised that fear of contact and negative attitudes towards nursing students were reduced via training. Appelbaum et al. (2020) [41] emphasised the importance of more respectful teamwork, team effectiveness, and team cohesion, which are characterised by power distance, equality, and psychological safety. In this regard, our study also showed that a respectful negotiation process was followed in interventions in order to overcome challenges in paediatric emergencies. Cheng et al. (2007) [42] confirmed that a negative attitude towards interprofessional interactions between medical and nursing students can be improved through training. This is also in line with the study by King et al. (2013) [37], where improved collaboration was demonstrated using the example of respiratory therapy.

Furthermore, our results indicate the development of a positive interprofessional relationship between nursing and medical students through joint simulation training.

The qualitative analysis showed that a predefined leadership role contributes to a team incorporating the time-out method significantly more often. This improves patient safety, as team members are simultaneously updated on all interventions and nursing and medical students can contribute hypotheses and other aspects to address a given emergency situation [43,44]. Aggarwal et al. (2014) [45] confirmed that there was an increase in patient safety through simulation training. Our study’s qualitative data suggest that simulations exhibit more orderliness when a predetermined management approach is in place. Thomas et al. (2007) [46] also came to the same conclusion and confirmed that the teaching teamwork used in neonatal resuscitation leads to more frequent team-oriented behaviour and that speaking up also depends on the hierarchical structure of a team. Fang et al. (2018) [47] confirmed that the team time-out initiative guarantees a high level of patient safety. Clerihew et al. (2022) [1] pointed out that patient safety in paediatric emergencies must be considered, particularly in the context of the direct responsibility of front-line professionals. Sharma et al. (2023) [48] investigated simulation-based basic life support training in a cross-sectional study, with the results showing a significant increase in the correct application of cardiopulmonary resuscitation. Kodikara (2022) [49] came to a similar conclusion, particularly for undergraduate medical education, including a guide for medical teachers.

The primary focus in high-acuity paediatric cardiac intensive care units is on achieving optimal staff performance and refining resuscitation skills [50,51]. In our study, we were also able to demonstrate that the application of immediate life-saving measures and guidelines, such as the ABCDE algorithm of the European Resuscitation Council, was a helpful skill [52]. Appelbaum et al. (2020) [41] confirmed that psychological safety and power distance had an impact on team cohesion and the effectiveness of collaboration. Collaboration between nurses and medical students can help to develop interventions to improve relationships after graduation [41]. In addition, Wai et al. (2021) [38] confirmed the promotion of interprofessional teamwork between nursing and medical students through simulation training as traditional hierarchical power structures between nurses and doctors were reduced. In addition, a flat hierarchy is also characterised by the fact that team members act with greater self-confidence without the fear of saying something wrong [53,54]. In our study, task management was exemplified by a structured lead function, the designation of a lead before simulation training commenced, assuming control of the situation, and effective workload distribution. In addition, knowing the work environment, planning ahead, and requesting help early (triggering a cardiac alarm) were outcomes that were evident in the video analysis [45,55]. Satisfactory levels of effective and safe communication by both nursing and medical students could not always be observed in the individual scenarios. Visser et al. (2017) [56] investigated the perceptions of medical and nursing students about interprofessional simulation training, with the result showing that willingness to participate in interprofessional education is influenced by obstacles, perceptions, and attitudes towards nurses. The students also made efforts to effectively mobilise resources, make decisions, and distribute orders through intentional delegation [57]. An essential finding in analysing the complex contexts of paediatric emergencies is the interrelation, complementarity, and occasional indistinct boundaries between individual communication strategies.

## 5. Limitations

There are several limitations of this study. (1) The quantitative sample could have had a larger sample size to make the data even more comparable. (2) Generally valid conclusions and the transferability of the results are only possible to a limited extent. (3) Another limitation is our reliance on the self-reported assessments via questionnaires. (4) The simulation training courses exclusively concentrate on paediatric emergencies. (5) Therefore, only limited conclusions can be drawn for other emergency scenarios, both in terms of quantitative and qualitative results. (6) An additional limitation arose because the selected roles were also taken on by teachers. This may have caused student uncertainty. (7) Another limitation is the risk of the distortion due to the reporting of selective results.

## 6. Conclusions

This study presents substantial evidence of the learning impact of interprofessional paediatric simulation training among nursing and medical students.

Interprofessional simulation significantly enhanced attitudes towards interprofessional learning in both student groups. The students highly rated and appreciated the course, with the medical students having a better overall attitude towards nursing students than the latter had towards the former. Despite receiving identical training, the communication strategies taught were used very differently and in different contexts. In scenarios where there was a clear lead, the strategies were more effective. A flat hierarchy and a clear lead are crucial for successful simulations of a critically ill child. 

Interprofessional high-fidelity simulation training has a decisive influence on the development of future professional identity but also on the development of a professional understanding of the role of working in a team. Additional benefits of simulation training were described herein, including a safe and risk-free learning environment and the ability of a multidisciplinary team to train with nursing and medical students.

## Figures and Tables

**Table 1:** Characteristics of the students surveyed (n = 76).

		Medicine		Nursing		Total	
		(n = 41)		(n = 35)		(n = 76)	
		n	%	n	%	n	%
Gender							
	female	30	73%	25	71%	55	72%
	male	11	27%	10	29%	21	28%
Age							
	21–23	13	32%	17	49%	30	39%
	24–26	20	49%	10	29%	30	39%
	27–30	4	10%	4	11%	8	11%
	31+	4	10%	4	11%	8	11%
Academic year							
	1	1	2.4%	0	0.0%	1	1%
	2	0	0.0%	0	0.0%	0	0%
	3	8	19.5%	34	97.1%	42	55%
	4	13	31.7%	0	0.0%	13	17%
	5	16	39.0%	0	0.0%	16	21%
	6	3	7.3%	1	2.9%	4	5%

**Table 2:** Overview change pre- and post-simulation in both groups (n = 76). Significance values and effect size are given.

		Pre		Post					
	Scales for Nursing and Medical Students	M	SD	M	SD	n	z	p	r
Communication and Teamwork (Subscale UWE-IP_german)									
	Medicine	18.2	2.9	17.8	3.8	41	−0.939	0.348	
	Nursing	18.7	4.3	17.7	3.7	35	−2.428	0.015 *	−0.41
Interprofessional Learning (Subscale UWE-IP_german)									
	Medicine	14.6	4.2	13.1	4	40	−3.304	<0.001 ***	−0.52
	Nursing	17.4	4.9	14.5	4.7	35	−3.991	<0.001 ***	−0.67
Interprofessional Interaction (Subscale UWE-IP_german)									
	Medicine	30.8	4.4	29	5	41	−2.621	0.009 **	−0.17
	Nursing	31.9	3.8	30.1	7	35	−1.71	0.087	
Interprofessional Relationship (Subscale UWE-IP_german)									
	Medicine	18.3	3.8	17.1	3.5	41	−2.472	0.013 *	−0.39
	Nursing	19.2	4.6	18.1	5	35	−1.682	0.093	
Attitude towards the other professional group (Subscale Greif Mie)									
	Medicine	94.1	10.3	95.9	8.4	40	2.249	0.025 *	0.36
	Nursing	88.2	9.4	89.6	12.7	34	0.991	0.322	

For UWE-IP, the lower the value, the better, and the values have a range of 1. Subscale: 9–36; subscales 2–3: 9–45; and subscale 4: 8–40. Greif Mie: 0–100 (the higher, the better). Note: * *p* < 0.05 **; *p* < 0.01; and *** *p* < 0.001.

**Table 3:** Number of communication strategies employed in the three quality groups.

Frequency of the following Communication Strategies	Quality of the Scenarios as Assessed by Teachers					
	Very Good (1)		Moderate (2)		Poor (3)	
	M	SD	M	SD	M	SD
Closed-loop communication	7.3	8	6.3	6.4	3.3	3.6
Speaking up	3.4	3.1	7.8	10.2	4.3	3
10 s for 10 min	1.3	1.2	1.2	1.3	0.5	0.6

## Data Availability

The data presented in this study are available on request from the corresponding author.

## Public Involvement Statement

There was no public involvement in any aspect of this research.

## Guidelines and Standards Statement

This manuscript was drafted against the quality of mixed methods studies in health services research (GRAMMS) for mixed methods studies in health services research.
